# Observations on the Peculiar Diseases to Which the Famine of Last Year Gave Origin, and on the Morbid Effects of Insufficient Nourishment

**Published:** 1848-06

**Authors:** Daniel Donovan

**Affiliations:** Skibbereen


*Observations on the peculiar Diseases to which the Famine of last year gave origin, and on the Morbid Effects of insufficient Nourishment.* By Daniel Donovan, M.D., Skibbereen.—In the above district, whose name has become familiar to every one, famine and pestilence broke out earlier, raged more severely, and committed greater ravages than in any other part of the kingdom. Dr. Donovan's ample opportunities of observation here brought him in contact with many forms of disease new to him, and undescribed by medical authors. These he proposes to designate famine cachexia, or lingering starvation; famine fever; famine dysentery; land scurvy; anemic dropsy; stomatigus and pemphigus malignus; abortion and asthenic sterility. Although at all times cases of death occur in Ireland which are traceable to insufficient nourishment, few persons previous to last year had seen human beings die from absolute want of food. In most cases, even those who died of starvation could procure some food, which preserved life until exposure to cold or some other accidental cause extinguished the feeble spark. In many instances diarrhœa, asphyxia, or syncope preceded death, and thus in many cases the death may have been attributed to disease, although it was, in reality, the result of imperfect alimentation.

*Symptoms of Starvation.*—The persons who have suffered from starvation describe the pain of hunger as at first being very acute, but after want of food for twenty-four hours, the pain subsides, and is succeeded by a feeling of weakness and sinking, especially felt in the epigastric region, with insatiable thirst and distressing feeling of coldness over the whole body. In a short time the face and limbs become frightfully emaciated; the eyes acquire a most peculiar stare; the skin exhales a peculiar and offensive fetor; and is covered with a brownish filthy looking coating, almost as indelible as varnish. Dr. Donovan first supposed this to be encrusted filth, but was subsequently convinced that it was a peculiar secretion poured out by the exhalants of the surface. The sufferer tottered in walking like a drunken man, his voice became weak as in cholera, he whined like a child, and burst into tears on the slightest occasion. Prostration of the mental faculties kept pace with the physical debility. Imbecility, and sometimes complete idiotism were observed; but in no case the mania or delirium described in many instances of starvation in shipwrecks.

The case of a boy aged fourteen is described, who cut the throats of two other children to get possession of some Indian meal, and who, when subsequently seen by Dr. Donovan, appeared like a congenital idiot. His faculties have again brightened since he was committed to jail, and has been supplied with food. [It would be interesting, in a medico-legal point of view, to know whether the boy was tried for this crime.] In the young and infant population the natural instincts seemed to be sharpened by the same causes which paralysed the faculties of the adult—babes scarcely able to speak became expert beggars, and infants a few weeks old would drink greedily from any vessel, and attempt to feed themselves.

*Post-mortem appearances.*—Acccording to Dr. Donovan the post-mortem appearances are in no respect peculiar or characteristic. They are in kind similar to those which may accompany various lingering diseases; and their true cause is to be inferred rather from the absence of any pathological indications of other diseases, than from any characteristic peculiarities. Dr. Donovan never met with inflammation or erosion of the stomach, described as common in cases of starvation. He supposes that these may occur in persons in health who have been suddenly deprived of food, and he is induced to ascribe it to the action of the gastric juice.—[We doubt this. Erosion by the gastric juice takes place where food has been taken shortly before death. It is not secreted in fasting animals.]

The appearances witnessed were excessive emaciation, total absorption of the fat on the surface of the body, total disappearance of the omentum and a peculiarly thin condition of the small intestines, which, in such cases were so transparent, that if the deceased had swallowed food immediately before death, the contents could be seen through the coats of the bowel. In one case Dr. Donovan was able to recognise a piece of raw green cabbage in the duodenum of a man who had died of want. Dr. Donovan regards this condition of the intestines as the strongest proof of starvation. The gall-bladder was commonly full. The urinary bladder contracted and empty. The heart pale, soft, and flabby. The brain and lungs normal.

*Treatment.*—Dr. Donovan very properly points out the importance of a cautious return to the use of food. Too much given at once induces vomiting and exhaustion; a stimulating diet, too long continued, is apt to produce reaction terminating in fever. He found bread and milk, given frequently in small quantities, to answer best. Rice and soups aggravated the diarrhœa which is the most harassing symptom. It was best combatted by opium, decoction of logwood, and chalybeates.—*Dublin Medical Press.*

